# Supplementary material for: The Effects of Different Cotton Varieties on the Growth and Feeding Preferences of Helicoverpa armigera
Source: Insects. 2025 Jan 24;16(2):115. doi: 10.3390/insects16020115 (PMC11855669; doi:10.3390/insects16020115)
Supplement: Supplementary file 1 [file insects-16-00115-s001.zip › insects-3393795-Supplementary.pdf]

The mean, standard deviation, significance level, F-value, P-value, and degrees of freedom are delineated in the ensuing table. Different lowercase letters indicated that there were significant differences among different varieties by Duncan multiple comparison (*P* < 0.05). In the data table, df1 represents inter-group degrees of freedom, df2 represents intra-group degrees of freedom. In the subsequent table, a P-value of 0.000 signifies that the P-value is less than 0.001. The same applies below.

Table S1-I In the selective experiment, the feeding area of *H. armigera* for 5 varieties of cotton

Treatment groups of different varieties	Mean (cm^2)	SD (cm^2)	Significance ($P < 0.05$)	F-value	P-value	df1	df2
A	0.4916	0.0541	a	5.892	0.003	4	20
B	0.3953	0.0442	c	5.892	0.003	4	20
C	0.4599	0.0256	ab	5.892	0.003	4	20
D	0.4902	0.0338	a	5.892	0.003	4	20
E	0.4073	0.0456	bc	5.892	0.003	4	20

Table S1-II In the non-selective experiment, the feeding area of *H. armigera* for 5 varieties of cotton

Treatment groups of different varieties	Mean (cm^2)	SD (cm^2)	Significance ($P < 0.05$)	F-value	P-value	df1	df2
A	0.5309	0.0155	a	9.993	0.002	4	10
B	0.4032	0.0244	c	9.993	0.002	4	10
C	0.4468	0.0429	bc	9.993	0.002	4	10
D	0.4709	0.0108	b	9.993	0.002	4	10
E	0.4082	0.0360	c	9.993	0.002	4	10

Table S1-III Mortality of *H. armigera* after feeding on different varieties of cotton leaves for 20 days

Treatment groups of different varieties	Mean (%)	SD (%)	Significance ($P < 0.05$)	F-value	P-value	df1	df2
A	21.00	4.18	c	4.145	0.013	4	20
B	33.00	5.70	a	4.145	0.013	4	20
C	28.00	5.70	abc	4.145	0.013	4	20
D	25.00	5.00	bc	4.145	0.013	4	20
E	31.00	5.48	ab	4.145	0.013	4	20

Table S2-I The weights of the 3rd-instar larvae of *H. armigera* after feeding on five varieties of cotton

Treatment groups of different varieties	Mean (mg)	SD (mg)	Significance ($P < 0.05$)	F-value	P-value	df1	df2
A	18.79	0.83	a	20.240	0.000	4	20
B	15.60	0.61	b	20.240	0.000	4	20
C	18.42	0.65	a	20.240	0.000	4	20
D	18.59	0.73	a	20.240	0.000	4	20
E	16.21	0.86	b	20.240	0.000	4	20

Table S2-II The weights of the 4th-instar larvae of *H. armigera* after feeding on five varieties of cotton

Treatment groups of different varieties	Mean (mg)	SD (mg)	Significance (P < 0.05)	F-value	P-value	df1	df2
A	37.93	1.37	a	11.740	0.000	4	20
B	31.14	1.12	b	11.740	0.000	4	20
C	35.56	2.26	a	11.740	0.000	4	20
D	36.89	2.57	a	11.740	0.000	4	20
E	32.22	1.91	b	11.740	0.000	4	20

Table S2-III The weights of the 5th-instar larvae of *H. armigera* after feeding on five varieties of cotton

Treatment groups of different varieties	Mean (mg)	SD (mg)	Significance (P < 0.05)	F-value	P-value	df1	df2
A	93.67	2.59	a	3.446	0.027	4	20
B	83.93	6.50	b	3.446	0.027	4	20
C	91.39	3.68	a	3.446	0.027	4	20
D	92.76	6.02	a	3.446	0.027	4	20
E	87.60	4.48	ab	3.446	0.027	4	20

Table S2-IV The weights of the 6th-instar larvae of *H. armigera* after feeding on five varieties of cotton

Treatment groups of different varieties	Mean (mg)	SD (mg)	Significance (P < 0.05)	F-value	P-value	df1	df2
A	264.78	13.66	a	22.330	0.000	4	20
B	206.03	7.12	d	22.330	0.000	4	20
C	239.73	10.26	b	22.330	0.000	4	20
D	251.03	11.83	ab	22.330	0.000	4	20
E	222.19	10.97	c	22.330	0.000	4	20

Table S2-V The durations of 3rd-instar *H. armigera* after feeding on 5 varieties of cotton

Treatment groups of different varieties	Mean (d)	SD (d)	Significance (P < 0.05)	F-value	P-value	df1	df2
A	5.79	0.14	b	2.844	0.051	4	20
B	6.43	0.28	a	2.844	0.051	4	20
C	5.89	0.50	ab	2.844	0.051	4	20
D	5.80	0.42	b	2.844	0.051	4	20
E	6.27	0.49	ab	2.844	0.051	4	20

Table S2-VI The durations of 4th-instar *H. armigera* after feeding on 5 varieties of cotton

Treatment groups of different varieties	Mean (d)	SD (d)	Significance (P < 0.05)	F-value	P-value	df1	df2
A	5.28	0.25	c	8.752	0.000	4	20
B	6.12	0.30	a	8.752	0.000	4	20
C	5.57	0.45	bc	8.752	0.000	4	20
D	5.31	0.51	c	8.752	0.000	4	20
E	5.95	0.36	ab	8.752	0.000	4	20

Table S2-VII The durations of 5th-instar *H. armigera* after feeding on 5 varieties of cotton

Treatment groups of different varieties	20	SD (d)	Significance (P < 0.05)	F-value	P-value	df1	df2
A	4.37	0.18	c	4.786	0.007	4	20
B	5.07	0.23	a	4.786	0.007	4	20
C	4.58	0.29	bc	4.786	0.007	4	20
D	4.45	0.30	c	4.786	0.007	4	20
E	4.87	0.18	ab	4.786	0.007	4	

Table S2-VIII The durations of 6th-instar *H. armigera* after feeding on 5 varieties of cotton

Treatment groups of different varieties	Mean (d)	SD (d)	Significance (P < 0.05)	F-value	P-value	df1	df2
A	5.79	0.14	b	7.422	0.001	4	20
B	6.43	0.28	a	7.422	0.001	4	20
C	5.89	0.50	ab	7.422	0.001	4	20
D	5.80	0.42	b	7.422	0.001	4	20
E	6.27	0.49	ab	7.422	0.001	4	20

Table S3-I The prepupal periods of *H. armigera* after feeding on five different cottons

Treatment groups of different varieties	Mean (d)	SD (d)	Significance (P < 0.05)	F-value	P-value	df1	df2
A	5.44	0.22	b	5.429	0.004	4	20
B	6.19	0.20	a	5.429	0.004	4	20
C	5.61	0.29	b	5.429	0.004	4	20
D	5.54	0.54	b	5.429	0.004	4	20
E	6.04	0.19	a	5.429	0.004	4	20

Table S3-II The pupation rates of *H. armigera* after feeding on five different cottons

Treatment groups of different varieties	Mean (%)	SD (%)	Significance (P < 0.05)	F-value	P-value	df1	df2
A	79.00	4.18	a	4.145	0.013	4	20
B	67.00	5.70	c	4.145	0.013	4	20
C	72.00	5.70	abc	4.145	0.013	4	20
D	75.00	5.00	ab	4.145	0.013	4	20
E	69.00	5.48	bc	4.145	0.013	4	20

Table S3-III The pupal malformation rates of *H. armigera* after feeding on five different cottons

Treatment groups of different varieties	Mean (%)	SD (%)	Significance (P < 0.05)	F-value	P-value	df1	df2
A	20.21	1.96	b	4.537	0.009	4	20
B	30.13	6.48	a	4.537	0.009	4	20
C	22.37	3.83	b	4.537	0.009	4	20
D	21.23	4.95	b	4.537	0.009	4	20
E	29.13	5.89	a	4.537	0.009	4	20

Table S3-IV The female ratios of *H. armigera* after feeding on five different cottons

Treatment groups of different varieties	Mean (%)	SD (%)	Significance (P < 0.05)	F-value	P-value	df1	df2
A	51.84	3.64	a	0.406	0.802	4	20
B	49.24	4.71	a	0.406	0.802	4	20
C	51.35	6.28	a	0.406	0.802	4	20
D	52.18	6.45	a	0.406	0.802	4	20
E	49.04	4.34	a	0.406	0.802	4	20

Table S3-V The female pupal weights of *H. armigera* after feeding on five different cottons

Treatment groups of different varieties	Mean (mg)	SD (mg)	Significance (P < 0.05)	F-value	P-value	df1	df2
A	252.84	8.58	a	31.493	0.000	4	20
B	203.76	3.96	c	31.493	0.000	4	20
C	232.77	4.75	b	31.493	0.000	4	20
D	248.56	9.89	a	31.493	0.000	4	20
E	214.57	12.14	c	31.493	0.000	4	20

Table S3-VI The male pupal weights of H. armigera after feeding on five different cottons

Treatment groups of different varieties	Mean (mg)	SD (mg)	Significance (P < 0.05)	F-value	P-value	df1	df2
A	245.76	9.09	a	29.589	0.000	4	20
B	193.25	6.63	c	29.589	0.000	4	20
C	219.38	9.52	b	29.589	0.000	4	20
D	236.14	11.73	a	29.589	0.000	4	20
E	203.48	7.00	c	29.589	0.000	4	20

Table S3-VII The female pupal stages of *H. armigera* after feeding on five different cottons

Treatment groups of different varieties	Mean (d)	SD (d)	Significance (P < 0.05)	F-value	P-value	df1	df2
A	8.74	0.29	c	5.873	0.003	4	20
B	10.07	0.52	a	5.873	0.003	4	20
C	9.69	0.48	ab	5.873	0.003	4	20
D	9.07	0.62	bc	5.873	0.003	4	20
E	9.82	0.59	a	5.873	0.003	4	20

Table S3-VIII The male pupal stages of *H. armigera* after feeding on five different cottons

Treatment groups of different varieties	Mean (d)	SD (d)	Significance (P < 0.05)	F-value	P-value	df1	df2
A	8.94	0.49	c	9.000	0.000	4	20
B	10.49	0.68	a	9.000	0.000	4	20
C	9.10	0.46	c	9.000	0.000	4	20
D	9.00	0.30	c	9.000	0.000	4	20
E	9.76	0.46	b	9.000	0.000	4	20

Table S4-I Eclosion rate of *H. armigera* after feeding on five different cottons

Treatment groups of different varieties	Mean (%)	SD (%)	Significance ($P < 0.05$)	F-value	P-value	df1	df2
A	73.46	6.60	a	8.455	0.000	4	20
B	55.27	7.49	b	8.455	0.000	4	20
C	71.88	6.76	a	8.455	0.000	4	20
D	72.25	7.21	a	8.455	0.000	4	20
E	59.45	3.76	b	8.455	0.000	4	20

Table S4-II The adult deformity rate of *H. armigera* after feeding on five different cottons

Treatment groups of different varieties	Mean (%)	SD (%)	Significance ($P < 0.05$)	F-value	P-value	df1	df2
A	29.28	3.22	b	8.217	0.000	4	20
B	42.70	6.00	a	8.217	0.000	4	20
C	32.83	3.10	b	8.217	0.000	4	20
D	29.91	5.91	b	8.217	0.000	4	20
E	41.07	5.50	a	8.217	0.000	4	20

Table S4-III The number of eggs laid by *H. armigera* after feeding on five different cottons

Treatment groups of different varieties	Mean (egg)	SD (egg)	Significance ($P < 0.05$)	F-value	P-value	df1	df2
A	628.67	37.78	a	1.452	0.287	4	10
B	532.80	64.03	a	1.452	0.287	4	10
C	584.60	36.23	a	1.452	0.287	4	10
D	593.27	89.90	a	1.452	0.287	4	10
E	544.13	25.29	a	1.452	0.287	4	10

Table S4-IV The hatching rate of *H. armigera* after feeding on five different cottons

Treatment groups of different varieties	Mean (%)	SD (%)	Significance ($P < 0.05$)	F-value	P-value	df1	df2
A	71.67	5.03	a	36.983	0.000	4	10
B	43.33	4.93	c	36.983	0.000	4	10
C	63.33	2.08	b	36.983	0.000	4	10
D	66.67	2.52	ab	36.983	0.000	4	10
E	45.67	2.52	c	36.983	0.000	4	10

Table S4-V The longevity of female adults of *H. armigera* after feeding on five different cottons

Treatment groups of different varieties	Mean (d)	SD (d)	Significance ($P < 0.05$)	F-value	P-value	df1	df2
A	9.20	0.20	a	7.613	0.001	4	20
B	8.12	0.23	c	7.613	0.001	4	20
C	8.83	0.46	ab	7.613	0.001	4	20
D	9.02	0.55	a	7.613	0.001	4	20
E	8.38	0.24	bc	7.613	0.001	4	20

Table S4-VI The longevity of male adults of *H. armigera* after feeding on five different cottons

Treatment groups of different varieties	Mean (d)	SD (d)	Significance ($P < 0.05$)	F-value	P-value	df1	df2
A	8.20	0.61	a	15.382	0.000	4	20
B	6.61	0.40	b	15.382	0.000	4	20
C	7.73	0.31	a	15.382	0.000	4	20
D	7.96	0.37	a	15.382	0.000	4	20
E	6.80	0.25	b	15.382	0.000	4	20
